# *catena*-Poly[[bis(pyridine)­lead(II)]bis(μ-penta­fluoro­benzene­thiol­ato)]

**DOI:** 10.1107/S160053681101659X

**Published:** 2011-05-07

**Authors:** Sarah E. Appleton, Glen G. Briand, Andreas Decken, Anita S. Smith

**Affiliations:** a Department of Chemistry and Biochemistry, Mount Allison University, 63C York Street, Sackville, NB, Canada E4L 1G8; b Department of Chemistry, University of New Brunswick, Fredericton, NB, Canada E3B 5A3

## Abstract

The title compound, [Pb(C_6_F_5_S)_2_(C_5_H_5_N)_2_]_*n*_, shows the Pb^II^ atom in a ψ-trigonal bipyramidal S_2_N_2_ bonding environment. Pyridine N atoms occupy axial sites, while thiol­ate S atoms and a stereochemically active lone pair occupy equatorial sites. Very long inter­molecular Pb⋯S inter­actions [3.618 (4) and 3.614 (4) Å] yield a weakly associated one-dimensional polymeric structure extending parallel to [010].

## Related literature

Lead(II) thiol­ates tend to form polymeric structures in the solid state *via* inter­molecular Pb⋯S inter­actions, see: Davidovich *et al.* (2010 ▶) and references therein; Eichhöfer (2005 ▶). However, the bonding environment at lead and the degree of inter­molecular bonding may be altered *via* the introduction of Lewis base ligands that occupy metal coordination sites, see: Appleton *et al.* (2004 ▶); Briand *et al.* (2007 ▶). It has been shown that [(F_5_C_6_S)_2_Pb]_n_ exhibits a three-dimensional framework structure containing hexa­coordinated Pb^II^ atoms (Fleischer *et al.*, 2006 ▶). For van der Waals radii, see: Bondi (1964 ▶); Brown (1978 ▶). 
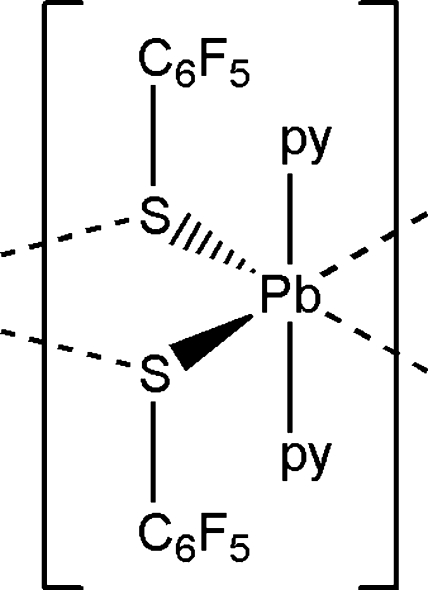


## Experimental

### 

#### Crystal data

[Pb(C_6_F_5_S)_2_C_5_H_5_N)_2_]
*M* *_r_* = 763.63
Monoclinic, 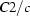

*a* = 19.9288 (19) Å
*b* = 5.0416 (5) Å
*c* = 24.9155 (19) Å
β = 111.339 (3)°
*V* = 2331.7 (4) Å^3^
*Z* = 4
Mo *K*α radiation
μ = 7.51 mm^−1^
*T* = 198 K
0.57 × 0.15 × 0.10 mm

#### Data collection

Bruker SMART1000/P4 diffractometer
Absorption correction: multi-scan (*SADABS*; Sheldrick, 2008*a* ▶) *T*_min_ = 0.099, *T*_max_ = 0.521
6756 measured reflections
2575 independent reflections
2421 reflections with *I* > 2σ(*I*)
*R*_int_ = 0.055

#### Refinement

*R*[*F* ^2^ > 2σ(*F* ^2^)] = 0.040
*wR*(*F* ^2^) = 0.097
*S* = 1.06
2575 reflections
168 parameters
H-atom parameters constrained
Δρ_max_ = 3.83 e Å^−3^
Δρ_min_ = −2.71 e Å^−3^

### 

Data collection: *SMART* (Bruker, 1999 ▶); cell refinement: *SAINT* (Bruker, 2006 ▶); data reduction: *SAINT*; program(s) used to solve structure: *SHELXS97* (Sheldrick, 2008*b* ▶); program(s) used to refine structure: *SHELXL97* (Sheldrick, 2008*b* ▶); molecular graphics: *SHELXTL* (Sheldrick, 2008*b* ▶); software used to prepare material for publication: *SHELXTL*.

## Supplementary Material

Crystal structure: contains datablocks I, global. DOI: 10.1107/S160053681101659X/hg5027sup1.cif

Supplementary material file. DOI: 10.1107/S160053681101659X/hg5027Isup2.cdx

Structure factors: contains datablocks I. DOI: 10.1107/S160053681101659X/hg5027Isup3.hkl

Additional supplementary materials:  crystallographic information; 3D view; checkCIF report

## Figures and Tables

**Fig. 1.**
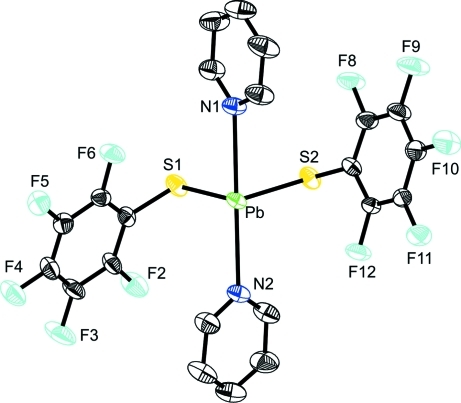
X-ray crystal structure of (I), with displacement ellipsoids drawn at the 50% probability level. H atoms have been omitted for clarity. Selected bond distances (Å) and angles (°): Pb—S(1) 2.650 (2), Pb—S(2) 2.653 (2), Pb—N(1) 2.643 (7), Pb—N(2) 2.637 (7), S(1)—Pb—S(2) 87.13 (6), S(1)—Pb—N(1) 91.44 (16), S(1)—Pb—N(2) 86.47 (15), S(2)—Pb—N(1) 86.69 (16), S(2)—Pb—N(2) 91.48 (16), N(1)—Pb—N(2) 177.29 (17).

**Fig. 2.**
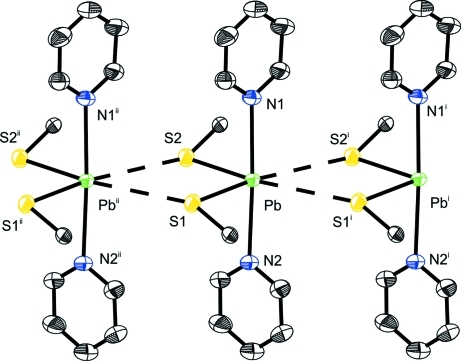
X-ray crystal structure of (I) showing the polymeric structure, with displacement ellipsoids drawn at the 50% probability level. All hydrogen atoms and C6F5 group carbon atoms (except α-carbon) have been omitted for clarity. Symmetry transformations used to generate equivalent atoms: (i) -1 + x, y, z; (ii) +1 + x, y, z.

## supplementary crystallographic information

### Comment

Lead(II) thiolates tend to form polymeric structures in the solid state *via* intermolecular Pb ··· S interactions (Davidovich *et al.*, 2010, and references therein; Eichhöfer, 2005). However, the bonding environment at lead and the degree of intermolecular bonding may be altered *via* the introduction of Lewis base ligands that occupy metal coordination sites (Appleton *et al.*, 2004; Briand *et al.*, 2007). It has been shown that [(F_5_C_6_S)_2_Pb]_n_ exhibits a three-dimensional layered structure containing hexacoordinated Pb^II^ atoms (Fleischer *et al.*, 2006). The corresponding bis-pyridine adduct (I) (Fig. 1) shows Pb1 in a ψ -trigonal bipyramidal bonding environment, with two pyridine nitrogen atoms in *trans* axial sites [N1—Pb—N2 = 177.29 (17)°] and two sulfur atoms in *cis* equatorial sites [S1—Pb—S2 = 87.13 (6)°]. The remaining "open" equatorial site is presumably occupied by the stereochemically active lone pair of Pb^II^. This is a similar bonding motif to that observed for (2,6-Me_2_C_6_H_3_S)_2_Pb × 2py (Appleton *et al.*, 2004), but shows some subtle structural differences. The Pb—N bond distances in (I) [Pb—N1 = 2.643 (7), Pb—N2 = 2.637 (7) Å] are significantly shorter than those in (2,6-Me_2_C_6_H_3_S)_2_Pb × 2py [2.689 (3) and 2.695 (3) Å], while the Pb—S distances [Pb—S1 = 2.650 (2), Pb—S2 = 2.653 (2) Å] are significantly longer [2.6078 (9) and 2.6079 (9) Å for (2,6-Me_2_C_6_H_3_S)_2_Pb × 2py]. This may be rationalized by considering the increased electron withdrawing ability of the C_6_F_5_ group in (I) *versus* the 2,6-Me_2_C_6_H_5_ group in (2,6-Me_2_C_6_H_3_S)_2_Pb × 2py. The result is an effective increase in the Lewis acidity at the Pb centre, and shorter Pb—N Lewis acid-base bonding interactions. Very weak intermolecular Pb ··· S interactions [Pb—S1^i^ = 3.618 (4), Pb—S2^i^ = 3.614 (4) Å; (i) -1 + *x*, *y*, *z*; sum of van der Waals' radii = 3.8 Å] (Bondi, 1964; Brown, 1978) between adjacent molecules in (I) yield a one-dimensional polymeric structure (Fig. 2). These contacts are nearly *trans* to the short Pb—S bonds [S1—Pb—S2^i^ = 166.75 (5)°, S2—Pb—S1^i^ = 166.83 (5)°], yielding a distorted octahedral bonding arrangement at Pb. This weakly associated polymeric structure differs from that of (2,6-Me_2_C_6_H_3_S)_2_Pb × 2py, which is monomeric in the solid-state. Further, the structure possesses no intramolecular Pb ··· F contacts such as those observed in [(F_5_C_6_S)_2_Pb]_n_ (Fleischer *et al.*, 2006).

### Experimental

Synthesis of (C_6_F_5_S)_2_Pb × 2py: A solution of pyridine (0.520 g, 6.57 mmol) in thf (3 ml) was added dropwise to a stirred solution of (C_6_F_5_S)_2_Pb (0.100 g, 0.165 mmol) in thf (5 ml) to give a cloudy pale green solution. The solution was stirred for 15 minutes and filtered. After 1 d at 25°C, colorless rod-like crystals of (I) were collected by suction filtration (0.100 g, 0.131 mmol, 79%). Anal. Calc. for C_21_H_10_F_10_N_2_PbS_2_: C, 34.60; H, 1.32; N, 3.67. Found: C, 34.47; H, 1.05; N, 3.64. Mp 262°C. See expt further details section for spectroscopic data.

### Refinement

Hydrogen atoms were placed in calculated positions with C–H distances fixed at 0.93 Å and *U*_iso_ values = 1.2 *U*_eq_ of the carrier C atom.

### Crystal data

**Table:**

[Pb(C_6_F_5_S)_2_C_5_H_5_N)_2_]	*F*(000) = 1440
*M**_r_* = 763.63	*D*_x_ = 2.175 Mg m^−^^3^
Monoclinic, *C*2/*c*	Mo *K*α radiation, λ = 0.71073 Å
Hall symbol: -C 2yc	Cell parameters from 5261 reflections
*a* = 19.9288 (19) Å	θ = 2.2–27.9°
*b* = 5.0416 (5) Å	µ = 7.51 mm^−^^1^
*c* = 24.9155 (19) Å	*T* = 198 K
β = 111.339 (3)°	Parallelepiped, colourless
*V* = 2331.7 (4) Å^3^	0.57 × 0.15 × 0.10 mm
*Z* = 4	

### Data collection

**Table:**

Bruker SMART1000/P4 diffractometer	2575 independent reflections
Radiation source: fine-focus sealed tube, K760	2421 reflections with *I* > 2σ(*I*)
graphite	*R*_int_ = 0.055
φ and ω scans	θ_max_ = 27.5°, θ_min_ = 4.2°
Absorption correction: multi-scan (*SADABS*; Sheldrick, 2008a)	*h* = −25→25
*T*_min_ = 0.099, *T*_max_ = 0.521	*k* = −6→6
6756 measured reflections	*l* = −30→32

### Refinement

**Table:**

Refinement on *F*^2^	Primary atom site location: structure-invariant direct methods
Least-squares matrix: full	Secondary atom site location: difference Fourier map
*R*[*F*^2^ > 2σ(*F*^2^)] = 0.040	Hydrogen site location: inferred from neighbouring sites
*wR*(*F*^2^) = 0.097	H-atom parameters constrained
*S* = 1.06	*w* = 1/[σ^2^(*F*_o_^2^) + (0.0608*P*)^2^] where *P* = (*F*_o_^2^ + 2*F*_c_^2^)/3
2575 reflections	(Δ/σ)_max_ = 0.001
168 parameters	Δρ_max_ = 3.83 e Å^−^^3^
0 restraints	Δρ_min_ = −2.70 e Å^−^^3^

### Special details

**Table:**

Experimental. Crystal decay was monitored by repeating the initial 50 frames at the end of the data collection and analyzing duplicate reflections.FT—IR (cm^-1^): 669 w, 702 m, 750 m, 825 vw, 856 s, 972 s, 1001 m, 1153 w, 1215 w, 1263 vw, 1444 s, 1477 *versus*, 1510 s, 1595 m, 1608 vw, 2341 m, 2360 s. FT-Raman (cm^-1^): 74 s, 101 *versus*, 175 vw, 201 vw, 268 *versus*, 317 vw, 372 vw, 387 w, 444 vw, 513 m, 584 w, 859 m, 1003 s, 1032 m, 1277 vw, 1393 m, 1636 *versus*, 3069 m. NMR data (thf-*d*_8_, p.p.m.): ^1^H NMR, δ = 7.36 (m, 4H, NC_5_*H*_5_), 7.77 (tt, 2H, ^3^*J* (^1^H-^1^H) = 8 Hz, ^4^*J* (^1^H-^1^H) = 2 Hz, NC_5_*H*_5_), 8.67 (m, 4H, NC_5_*H*_5_); ^13^C{^1^H} NMR, δ = 115.8 (tm, ^2^*J* (^13^C-^19^F) = 22 Hz, S*C*_6_F_5_), 124.2 (s, N*C*_5_H_5_), 136.7 (s, N*C*_5_H_5_), 137.1 (dm, ^1^*J* (^13^C-^19^F) = 245 Hz, S*C*_6_F_5_), 137.7 (dm, ^1^*J* (^13^C-^19^F) = 247 Hz, S*C*_6_F_5_), 148.4 (dm, ^1^*J* (^13^C-^19^F) = 226 Hz, S*C*_6_F_5_), 149.4 (s, N*C*_5_H_5_); ^19^F NMR, δ = -166.2 (m, SC_6_*F*_5_), -164.5 (m, SC_6_*F*_5_), -133.9 (m, SC_6_*F*_5_).
Geometry. All e.s.d.'s (except the e.s.d. in the dihedral angle between two l.s. planes) are estimated using the full covariance matrix. The cell e.s.d.'s are taken into account individually in the estimation of e.s.d.'s in distances, angles and torsion angles; correlations between e.s.d.'s in cell parameters are only used when they are defined by crystal symmetry. An approximate (isotropic) treatment of cell e.s.d.'s is used for estimating e.s.d.'s involving l.s. planes.
Refinement. Refinement of *F*^2^ against ALL reflections. The weighted *R*-factor *wR* and goodness of fit *S* are based on *F*^2^, conventional *R*-factors *R* are based on *F*, with *F* set to zero for negative *F*^2^. The threshold expression of *F*^2^ > σ(*F*^2^) is used only for calculating *R*-factors(gt) *etc*. and is not relevant to the choice of reflections for refinement. *R*-factors based on *F*^2^ are statistically about twice as large as those based on *F*, and *R*- factors based on ALL data will be even larger.

### Fractional atomic coordinates and isotropic or equivalent isotropic displacement parameters (Å^2^)

**Table:**

	*x*	*y*	*z*	*U*_iso_*/*U*_eq_	
Pb	0.0000	0.57741 (4)	0.2500	0.02457 (12)	
S1	−0.00240 (9)	0.9584 (2)	0.17593 (6)	0.0308 (3)	
F2	−0.1488 (2)	0.9328 (7)	0.0811 (2)	0.0491 (11)	
F3	−0.1937 (2)	0.5959 (8)	−0.0085 (2)	0.0643 (15)	
F4	−0.1038 (2)	0.2260 (7)	−0.02233 (17)	0.0513 (10)	
F5	0.03376 (19)	0.1979 (7)	0.05311 (15)	0.0416 (8)	
F6	0.0806 (2)	0.5350 (7)	0.14221 (17)	0.0404 (8)	
N1	0.1419 (3)	0.5895 (8)	0.2954 (3)	0.0333 (11)	
C1	−0.0321 (3)	0.7450 (9)	0.1159 (2)	0.0261 (10)	
C2	−0.1022 (3)	0.7539 (11)	0.0757 (2)	0.0324 (11)	
C3	−0.1263 (4)	0.5816 (11)	0.0295 (3)	0.0369 (14)	
C4	−0.0802 (4)	0.3944 (11)	0.0226 (3)	0.0341 (13)	
C5	−0.0113 (3)	0.3793 (11)	0.0607 (3)	0.0304 (12)	
C6	0.0123 (3)	0.5558 (9)	0.1063 (3)	0.0278 (11)	
C7	0.1855 (3)	0.7571 (12)	0.2823 (3)	0.0408 (13)	
H7	0.1652	0.8817	0.2534	0.049*	
C8	0.2590 (4)	0.7531 (14)	0.3097 (3)	0.0512 (17)	
H8	0.2880	0.8719	0.2994	0.061*	
C9	0.2891 (4)	0.5700 (12)	0.3528 (4)	0.052 (2)	
H9	0.3387	0.5646	0.3726	0.062*	
C10	0.2453 (4)	0.3984 (13)	0.3659 (4)	0.053 (2)	
H10	0.2645	0.2719	0.3946	0.063*	
C11	0.1719 (4)	0.4119 (11)	0.3363 (3)	0.0426 (16)	
H11	0.1423	0.2918	0.3455	0.051*	

### Atomic displacement parameters (Å^2^)

**Table:**

	*U*^11^	*U*^22^	*U*^33^	*U*^12^	*U*^13^	*U*^23^
Pb	0.02186 (16)	0.02276 (15)	0.02304 (18)	0.000	0.00096 (12)	0.000
S1	0.0382 (8)	0.0238 (6)	0.0243 (7)	−0.0012 (5)	0.0040 (6)	−0.0011 (5)
F2	0.036 (2)	0.054 (2)	0.045 (3)	0.0214 (16)	0.0000 (19)	−0.0131 (16)
F3	0.031 (2)	0.089 (3)	0.052 (3)	0.0185 (19)	−0.011 (2)	−0.030 (2)
F4	0.046 (2)	0.055 (2)	0.042 (2)	0.0075 (18)	0.0033 (18)	−0.0236 (18)
F5	0.0405 (19)	0.0421 (18)	0.042 (2)	0.0155 (16)	0.0150 (17)	−0.0039 (15)
F6	0.0235 (17)	0.050 (2)	0.038 (2)	0.0079 (15)	−0.0006 (16)	−0.0015 (16)
N1	0.022 (2)	0.034 (2)	0.037 (3)	0.0008 (17)	0.003 (2)	0.0018 (18)
C1	0.029 (2)	0.023 (2)	0.023 (3)	0.001 (2)	0.006 (2)	0.0051 (19)
C2	0.028 (2)	0.037 (3)	0.029 (3)	0.010 (2)	0.006 (2)	−0.004 (2)
C3	0.027 (3)	0.045 (3)	0.029 (3)	0.007 (2)	−0.002 (3)	−0.008 (2)
C4	0.034 (3)	0.038 (3)	0.027 (3)	0.003 (2)	0.008 (3)	−0.008 (2)
C5	0.031 (3)	0.032 (2)	0.029 (3)	0.010 (2)	0.012 (3)	0.001 (2)
C6	0.023 (3)	0.030 (3)	0.026 (3)	0.0005 (19)	0.004 (2)	0.0038 (19)
C7	0.033 (3)	0.040 (3)	0.041 (4)	−0.003 (3)	0.005 (3)	0.006 (3)
C8	0.033 (3)	0.054 (4)	0.061 (5)	−0.013 (3)	0.010 (3)	0.003 (3)
C9	0.027 (3)	0.056 (4)	0.061 (5)	−0.001 (3)	0.003 (3)	−0.001 (3)
C10	0.039 (4)	0.049 (4)	0.051 (5)	0.005 (3)	−0.005 (4)	0.009 (3)
C11	0.032 (3)	0.040 (3)	0.047 (4)	−0.002 (2)	0.005 (3)	0.010 (2)

### Geometric parameters (Å, °)

**Table:**

Pb—N1	2.636 (5)	C2—C3	1.382 (8)
Pb—N1^i^	2.636 (5)	C3—C4	1.371 (8)
Pb—S1^i^	2.6519 (14)	C4—C5	1.359 (9)
Pb—S1	2.6519 (14)	C5—C6	1.384 (8)
S1—C1	1.761 (5)	C7—C8	1.373 (8)
F2—C2	1.336 (6)	C7—H7	0.9300
F3—C3	1.334 (8)	C8—C9	1.378 (11)
F4—C4	1.345 (7)	C8—H8	0.9300
F5—C5	1.342 (6)	C9—C10	1.350 (12)
F6—C6	1.334 (7)	C9—H9	0.9300
N1—C11	1.326 (8)	C10—C11	1.380 (10)
N1—C7	1.335 (8)	C10—H10	0.9300
C1—C6	1.379 (8)	C11—H11	0.9300
C1—C2	1.392 (7)		
			
N1—Pb—N1^i^	177.34 (18)	F5—C5—C4	119.8 (5)
N1—Pb—S1^i^	86.55 (12)	F5—C5—C6	120.7 (5)
N1^i^—Pb—S1^i^	91.52 (12)	C4—C5—C6	119.5 (5)
N1—Pb—S1	91.52 (12)	F6—C6—C1	120.1 (5)
N1^i^—Pb—S1	86.55 (12)	F6—C6—C5	117.3 (5)
S1^i^—Pb—S1	87.18 (6)	C1—C6—C5	122.7 (5)
C1—S1—Pb	93.67 (16)	N1—C7—C8	122.8 (6)
C11—N1—C7	117.6 (6)	N1—C7—H7	118.6
C11—N1—Pb	115.3 (4)	C8—C7—H7	118.6
C7—N1—Pb	127.0 (4)	C7—C8—C9	118.7 (7)
C6—C1—C2	115.9 (5)	C7—C8—H8	120.6
C6—C1—S1	122.1 (4)	C9—C8—H8	120.6
C2—C1—S1	122.0 (4)	C10—C9—C8	118.7 (7)
F2—C2—C3	117.8 (5)	C10—C9—H9	120.6
F2—C2—C1	120.1 (5)	C8—C9—H9	120.6
C3—C2—C1	122.1 (5)	C9—C10—C11	119.6 (7)
F3—C3—C4	119.8 (5)	C9—C10—H10	120.2
F3—C3—C2	120.7 (5)	C11—C10—H10	120.2
C4—C3—C2	119.5 (6)	N1—C11—C10	122.5 (7)
F4—C4—C5	120.3 (5)	N1—C11—H11	118.7
F4—C4—C3	119.4 (6)	C10—C11—H11	118.7
C5—C4—C3	120.3 (5)		

Symmetry codes: (i) −*x*, *y*, −*z*+1/2.
